# Implementation of a registry for quality improvement

**DOI:** 10.1186/1748-5908-8-S1-S4

**Published:** 2013-04-19

**Authors:** Caitlin W Brennan, Patricia S Groves, Richard B Colletti

**Affiliations:** 1 Veterans Affairs National Quality Scholars Program, Louis Stokes Cleveland VA Medical Center, Cleveland, Ohio, 44106, USA; 2 Frances Payne Bolton School of Nursing, Case Western Reserve University, Cleveland, Ohio, 44106, USA; 3 Center for Organization, Leadership, and Management Research, VA Boston Healthcare System, Boston, Massachusetts, 02130, USA; 4 Veterans Affairs National Quality Scholars Program, Iowa City VA Medical Center, Iowa City, Iowa, 52246, USA; 5 University of Iowa College of Nursing, Iowa City, Iowa, 52242, USA; 6 Department of Pediatrics, University of Vermont College of Medicine, Burlington, Vermont, 05405, USA

## Presentation

Crohns’ Disease and ulcerative colitis, or Inflammatory Bowel Disease (IBD), are rare in the pediatric population. Thus, conducting research centered on improving the quality of pediatric IBD care can be challenging for individual pediatric gastroenterology centers. By pooling data and sharing results across 35 care centers nationally, the ImproveCareNow network provides the opportunity for conducting quality improvement (QI) research aimed at improving care and outcomes for pediatric patients with IBD and their families [1]. The network serves as a platform for innovation and the establishment of evidence-based practices for IBD care.

ImproveCareNow includes a registry database for clinical care, QI and research; a program to teach QI to each site’s QI team; and tools for QI interventions. The presentation at the Academy for Healthcare Improvement Methods meeting demonstrated how a registry can be a vital part of QI and QI research.

The ImproveCareNow network uses the Model for Improvement to provide more reliable, pro-active care to pediatric patients with IBD [2]. Continuous improvement activities include examining registry data through detailed reports and acting appropriately based on these data. The centers receive monthly performance reports on 15 key clinical measures, a data quality report, and two population management reports. Control charts, benchmarking data, and care stratification data allow clinicians to identify high-risk patients and drill down to the patient level of analysis. The network also provides QI education and support, including monthly webinars, twice-yearly in-person learning sessions, and tools for effective system redesign. Network staff includes the network, scientific, and project directors, project manager, project coordinators, and QI consultants. Each center has its own QI team (physician leader, nurse leader, and improvement coordinator) that enrolls patients, enters data collected for each visit into a web-based database, and carries out QI activities to improve the site’s care delivery system. The network has demonstrated significant improvements in IBD remission rates, with approximately 75% of patients in remission in 2011 compared to 50% of patients in remission in 2007.

The network recently received funding from the Agency for Healthcare Research and Quality (AHRQ) to enhance its capacity. Funding will facilitate the roll-out of innovative informatics techniques to automate data transfer from the electronic health record directly to the registry, which will eliminate the need for manual data entry. Automation will also improve data collection for QI activities, population management procedures, pre-visit planning, patient-reported outcomes, comparative effectiveness research, and research focused on effectiveness of new biological agents to treat IBD. These informatics enhancements may reduce the time and personnel costs for the new centers anticipated to join a planned, larger sustainable network across 170 centers nationwide with over 50,000 patients.

ImproveCareNow also received additional funding to create a Chronic Care Collaborative Network or C3N. The overall aim is to bring together stakeholders with the shared goal of improving patient self-management, clinical practice, and disease outcomes through innovative use of information technology and social media. Studying the value of the network, or how well it achieves better outcomes at lower costs, and conducting comparative effectiveness research are major priorities as the network moves toward becoming a C3N.

## Commentary

The ImproveCareNow network’s use of the Model for Improvement to develop a registry and infrastructure for improving care illustrates the intersection between QI and research. The network approach may be more cost effective than randomized controlled trial methods and facilitates answering research questions in a “real world” setting. It incorporates several QI principles, including collecting real time data to track and monitor clinic processes and patient outcomes, feeding patient- and clinic-specific data back to clinicians, and benchmarking data to compare clinics across the network. At the same time, ImproveCareNow incorporates some of the activities associated with research, such as generating new knowledge, establishing evidence-based practices, linking findings to practice, and disseminating findings in peer-reviewed publications and presentations at scientific meetings. The network also serves as an example of how clinician researchers can disseminate QI research in peer-reviewed journals and meet both goals of improving care provided to patients and conducting research. An example of another successful network similar to ImproveCareNow is the Vermont Oxford Network, which has established a confidential database of outcomes and a QI collaborative for member centers [3]. The Vermont Oxford Network has successfully expanded both nationally and internationally and the National Quality Forum has endorsed two of the network’s measures related to infection and screenings for retinopathy [3].

Like the Vermont Oxford Network, ImproveCareNow seeks to expand. Throughout that process, ImproveCareNow leaders will likely face challenges related to resolving variation across sites. For example, scaling up from the initial 8 sites to the current 35 centers may have included early adopters [4] who had sufficient buy-in and resources to successfully participate in the network. Late adopters [4] will potentially have different needs and may pose new challenges for implementation and sustainability compared to the early adopter centers. Ensuring adequate protection of patients’ health information involves managing potential legal matters related to differing policies for data sharing and Institutional Review Board (IRB) requirements across sites. Network leaders are in the process of creating a federated (or central) IRB for participants, in order to develop standardized procedures for sharing of protected health information and establish processes for legal relationships. The extent to which successful expansion of the registry is generalizable to adult populations is unclear. The complexity of co-morbid conditions of adult patient populations may be challenging to manage with a registry. Adult providers may encounter system barriers to registry use.

Funding for ImproveCareNow is currently derived mainly from annual participation fees paid by the centers and from grants. One financial solution includes collaboration with payers with regard to reforming payment structures to share savings derived from targeted cost reduction and improved outcomes, given that payers are likely to benefit financially from reductions in emergency room visits and inpatient admissions.

## Recommendations

There are several recommendations for creating and implementing a registry for QI. Registry creators should engage patients and families, end-users, and other stakeholders (clinicians, researchers, QI experts, statisticians, data managers, research coordinators) early and often. Collaboration among interprofessional experts is a key component to not only ensuring that the registry data are updated with the most current clinical data and are usable at the point of care, but also is vital to the long-term success of the registry. Similarly, developing close relationships with IRB leaders will assist with ensuring that registry processes are compliant with ethical and regulatory standards. In order to promote sustainability, registry creators should seek a variety of sources of funding, including thinking creatively about partnerships with payers.

The goal of the ImproveCareNow network is to improve care for patients with chronic IBD through collaboration among patients, families, clinicians, administrators, and scientists. Serving as a model for research, QI, and innovation, ImproveCareNow has demonstrated effectiveness in improving care and outcomes for patients with IBD and their families.
